# Patterning highly ordered arrays of complex nanofeatures through EUV directed polarity switching of non chemically amplified photoresist

**DOI:** 10.1038/srep22664

**Published:** 2016-03-15

**Authors:** Subrata Ghosh, V. S. V. Satyanarayana, Bulti Pramanick, Satinder K. Sharma, Chullikkattil P. Pradeep, Israel Morales-Reyes, Nikola Batina, Kenneth E. Gonsalves

**Affiliations:** 1 School of Basic Sciences, Indian Institute of Technology Mandi, Mandi – 175005, India; 2 School of Computing and Electrical Engineering, Indian Institute of Technology Mandi, Mandi 175005, India; 3 Depto. de Química, Lab. de Nanotecnología e Ingeniería Molecular, CBI, UAM-I, Mexico D.F., Mexico

## Abstract

Given the importance of complex nanofeatures in the filed of micro-/nanoelectronics particularly in the area of high-density magnetic recording, photonic crystals, information storage, micro-lens arrays, tissue engineering and catalysis, the present work demonstrates the development of new methodology for patterning complex nanofeatures using a recently developed non-chemically amplified photoresist (n-CARs) poly(4-(methacryloyloxy)phenyl)dimethylsulfoniumtriflate) (polyMAPDST) with the help of extreme ultraviolet lithography (EUVL) as patterning tool. The photosensitivity of polyMAPDST is mainly due to the presence of radiation sensitive trifluoromethanesulfonate unit (triflate group) which undergoes photodegradation upon exposure with EUV photons, and thus brings in polarity change in the polymer structure. Integration of such radiation sensitive unit into polymer network avoids the need of chemical amplification which is otherwise needed for polarity switching in the case of chemically amplified photoresists (CARs). Indeed, we successfully patterned highly ordered wide-raging dense nanofeatures that include nanodots, nanowaves, nanoboats, star-elbow etc. All these developed nanopatterns have been well characterized by FESEM and AFM techniques. Finally, the potential of polyMAPDST has been established by successful transfer of patterns into silicon substrate through adaptation of compatible etch recipes.

Thanks to the remarkable developments of nanotechnology in the last few decades which witness the amazing progress in improving the properties and efficiencies of many end products often used in our everyday life. One such development is in the area of micro- and nanoelectronics^1^,^2^,^3^. In this context, the advanced fabrication techniques have made the rapid miniaturization of devices and machines possible. This progress is largely due to downscaling of patterned transistors in integrated circuits (ICs) as patterning is one among those most important steps followed during electronics device fabrication. Among the various techniques that have been developed over the years to fabricate ICs for electronic devices, due to its high resolution and throughput capability, photolithography as the most promising top-down nanofabrication method has been the workhorse for large scale production of micro/nanoscale devices in industries^4^,^5^. While immersion optical lithography technique has been the dominant patterning technique in semiconductor industries, extreme ultraviolet lithography (EUVL) using 13.5 nm light source is being considered to be the most potential and cost-effective next generation lithography (NGL) candidate for patterning ultrafine nanofeatures with high resolution, and this technique is approaching well into the commercialization^6^,^7^,^8^,^9^,^10^.

In photolithography, typically a photoresist (PR) is used as the key soft material to transfer desired images onto the silicon substrate^11^,^12^,^13^. Different types of etching techniques are used to transfer these images ultimately into the semiconductor patterning layer. Therefore, PR plays an important role in determining the pattern resolution, line-edge roughness (LER) and line-width roughness (LWR) of the end patterns. Hence, concurrently with need for the development of EUVL exposure tools, there is a dire need for subsequent optimization and upgrading of photoresist materials for the successful implementation of next generation lithography (NGL) tools and techniques.

Although chemically amplified resists (CARs) have been the workhorses mainly because of their high sensitivity and resolution, they often suffer from the issues of post exposure instability and acid diffusion that ultimately affects the LER or LWR of transferred patterns^14^,^15^,^16^,^17^,^18^. To overcome these shortcomings, researchers have focused on the development of non-chemically amplified resists (n-CARs) with improved lithography performance and capable of patterning both isolated and dense nanopatterns to ensure high throughput production of equally isolated (IC logic gates) and dense areas of devices (dynamic random-access memory (DRAM), ferroelectric random-access memory (FRAM)) for future successful implementations of next generation lithography (NGL) technology nodes^19^,^20^,^21^,^22^,^23^,^24^,^25^,^26^,^27^,^28^,^29^,^30^,^31^,^32^,^33^.

Among the various nanopattrens that are generated in semiconductor industries, complex nanofeatures such as nanodots, nanopillars, nanorings etc., are of special interests as they find wide applications in high-density magnetic recording, photonic crystals, information storage, micro-lens arrays, tissue engineering, catalysis and many more to mention^34^,^35^,^36^,^37^,^38^,^39^,^40^,^41^,^42^,^43^. These potential applications have garnered much interests among researchers for new methodologies and materials for achieving much ordered dense arrays of complex nanofeatures. The present manuscript demonstrates the potential applications of our recently developed polymeric non-chemically amplified negative tone photoresist, polyMAPDST, for patterning highly regular 34 nm complex nanopatterns using EUVL. PolyMAPDST has been prepared from MAPDST (4-(methacryloyloxy)phenyl)dimethylsulfoniumtriflate) monomer which contains radiation sensitive trifluoromethanesulfonate group. This trifluoromethanesulfonate group undergoes decomposition upon exposure to radiation, and brings in changes in polarity in the exposed area as compared to unexposed area. Therefore, polyMAPDST does not require any chemical amplification, and is directly sensitive to radiation. Complex nanofeatures, patterned using EUV lithography technique, have been characterized using field emission scanning electron microscope (FESEM) and atomic force microscope (AFM). Finally, the usefulness of polyMAPDST has been successfully established by transferring features into silicon substrate.

## Results and Discussions

### Extreme Ultraviolet Lithography (EUVL) for nanopatterning

**Figure 1:**
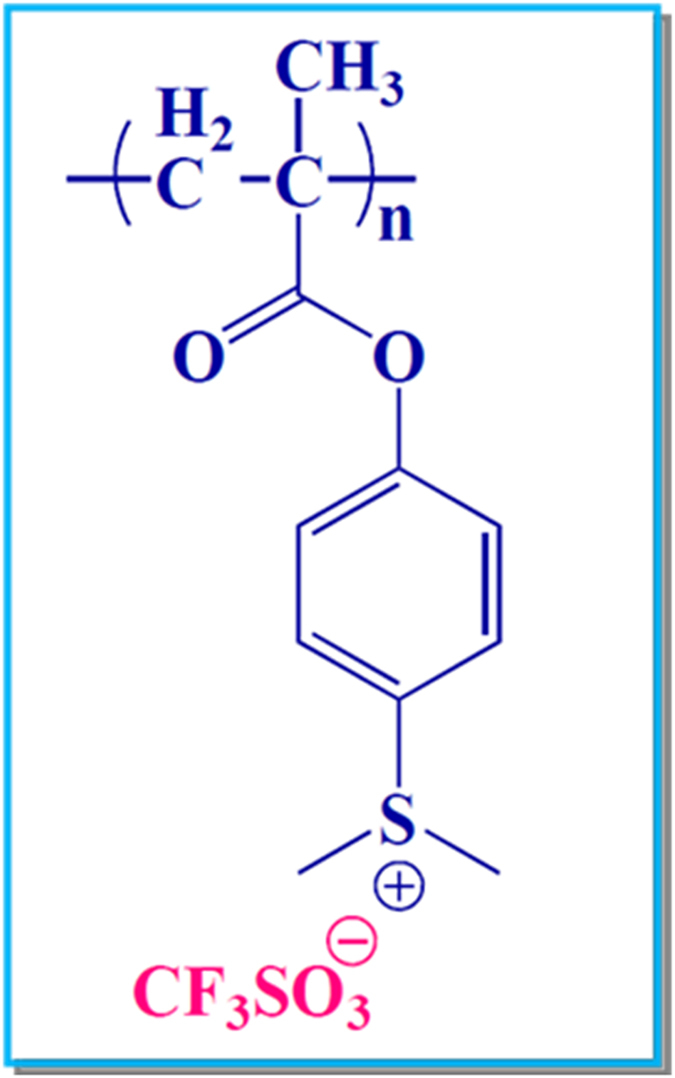
Chemical structure of polyMAPDST photoresist.

PolyMAPDST (Fig. 1) was synthesized following a four-step process^21^, and the end-product was obtained as white solid in good yields. The final product was characterized using various instrumental techniques that include FT-IR, NMR, TGA, DSC, and gel permeation chromatography (GPC). The characterization data were in good agreement with the reported data^21^.

**Figure 2:**
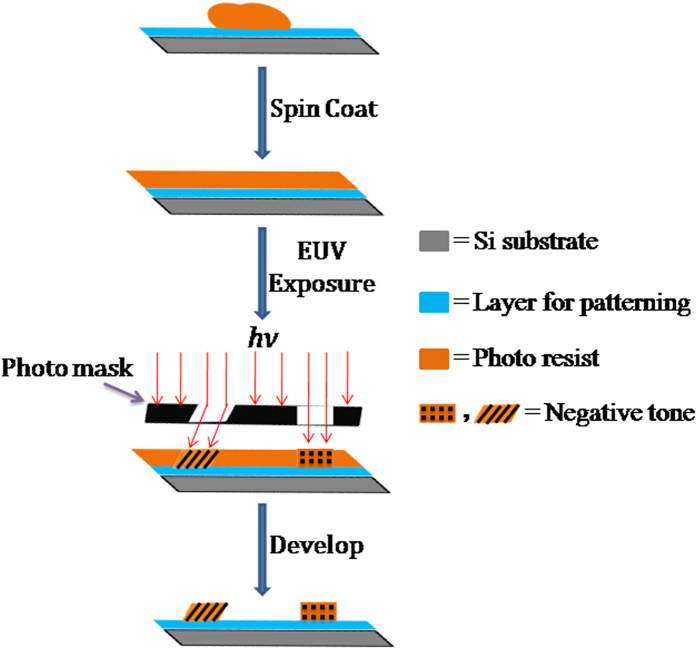
Schematic diagram of basic approach for nanopatterning using polyMAPDST.

Having pure photoresist polyMAPDST in hand, we started investigating its potential in patterning complex nanofeatures using EUV lithography. The resist solutions were prepared in methanol at 2% by weight in methanol and filtered by 0.2 micron teflon filter to remove large-sized particles in order to form uniform pinhole free resist film onto the silicon substrate. The filtered resist solution was spin coated at 5000 rpm speed for 60 sec onto HMDS treated 200 mm silicon wafer for around 35 nm thin films before EUV exposure evaluation. PolyMAPDST was prebaked at 115 °C for 90 sec. The center dose value was calculated to be 112.56 mJ/cm^2^. After optimizing the center dose value for polyMAPDST, the silicon substrates layered with photoresist were flood exposed with the exposure dose 88.19 mJ/cm^2^ using SEMATECH Berkeley Microfield Exposure Tool (MET) which is a high resolution EUV (13.5 nm) lithography tool. Samples were exposed using mask IMO228775 with Field of R4C3. After completion of EUV exposure, the exposed polyMAPDST layer was subjected to post exposure bake at 100 °C for 90 sec followed by developing of patterns in 0.002 N tetramethyl ammonium hydroxide (TMAH) solution in DI water (pH = 11.5) at room temperature for 15 sec. Then the developed patterns were rinsed with DI water for about 8 sec followed by drying with a mild flow of dry nitrogen gas (Fig. 2).

**Figure 3:**
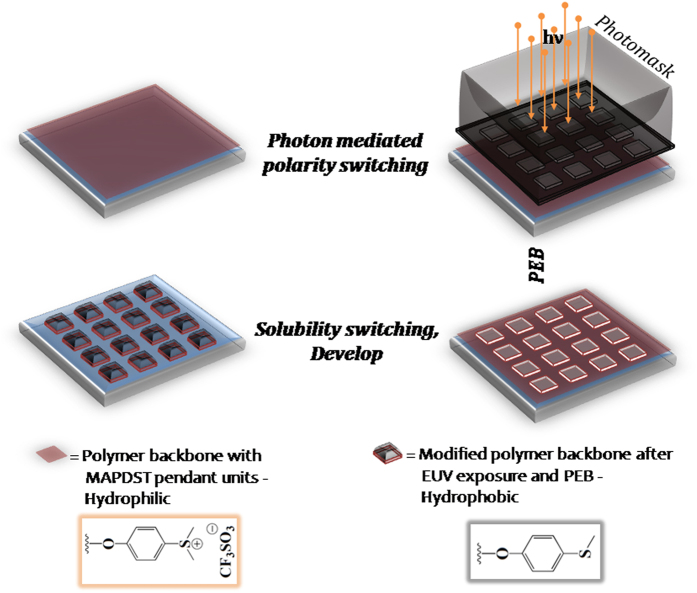
Designed polymer microstructure and solubility switching for EUV resist system.

As mentioned previously, the radiation sensitive functionality triflate unit undergoes photodegradation upon exposure with high energy EUV photon^21^,^13^, and ultimately the sulfonium (CH_3_-S^+^-CH_3_) group attached to phenyl ring of polyMAPDST gets converted to thioether (-S-CH_3_) functionality^21^,^23^. Therefore, the EUV mediated photodegradation process indeed induces polarity switching in the exposed area as compared to that of unexposed area by converting high polar hydrophilic sulfonium unit into nonpolar thioether functionality (Fig. 3) resulting in changes in their solubility in polar aqueous TMAH developer^21^. The exposed area remains undissolved whereas the unexposed area gets dissolved in TMAH solution; therefore, polyMAPDST acts as negative tone photoresist. It is worth noting that no external chemical amplification is necessary to bring in substantial change in polarity between exposed and unexposed area of polyMAPDST, and thus, it can be used as non-chemically amplified photoresists for patterning complex features.

**Figure 4:**
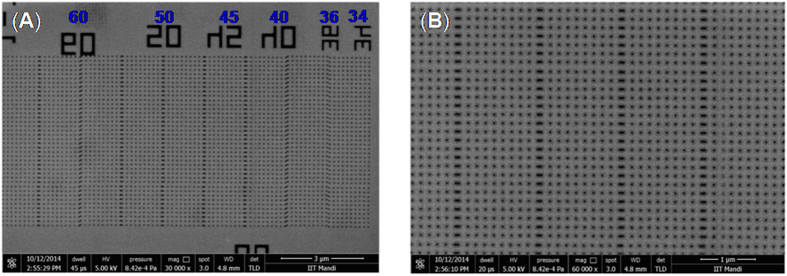
(**A**) FESEM images of highly dense arrays of 60, 50, 45, 40, 36 and 34 nm dots; (**B**) Higher magnification (60k) images of nanodots. (EUV dose 88.19 mJ/cm^2^).

**Figure 5:**
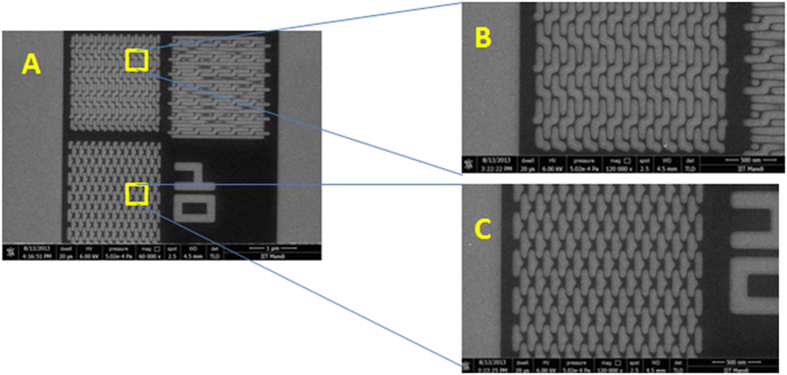
(**A**) FESEM images of EUV exposed nanowaves and nanoboates; (**B**) Higher magnification images (magnification: 120 k) of nanowaves; (**C**) Higher magnification images (magnification: 120 k) of nanowaves and nanoboats. (EUV dose 88.19 mJ/cm^2^).

The developed nanopatterns were characterized first using field emission scanning electron microscope (FESEM) which revealed the potential of polyMAPDST in patterning highly ordered dense nanodots, nanowaves, nanoboats, and star-elbows with sizes ranging from 34 nm to100 nm (Figs 4 and 5). Fascinatingly, the pattern edges were observed to be very sharp and devoid of blurring. This is a critical parameter for 3-D patterning particularly in the area of micro/nanoelectronics.

#### AFM Characterization

**Figure 6:**
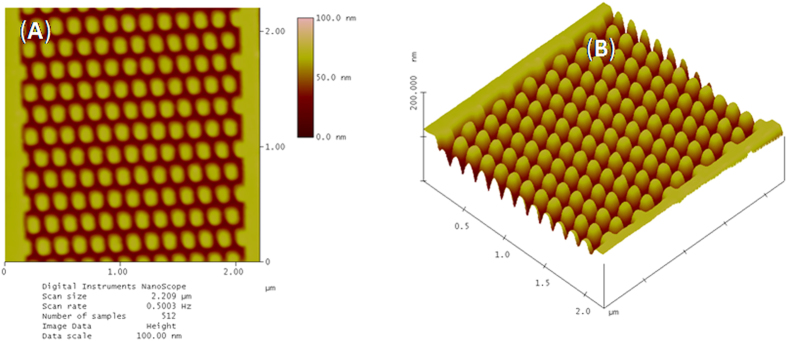
(**A**) AFM images of 34 nm dot features; (**B**) 3-D view of highly ordered nanodots. (EUV dose 88.19 mJ/cm^2^).

**Figure 7:**
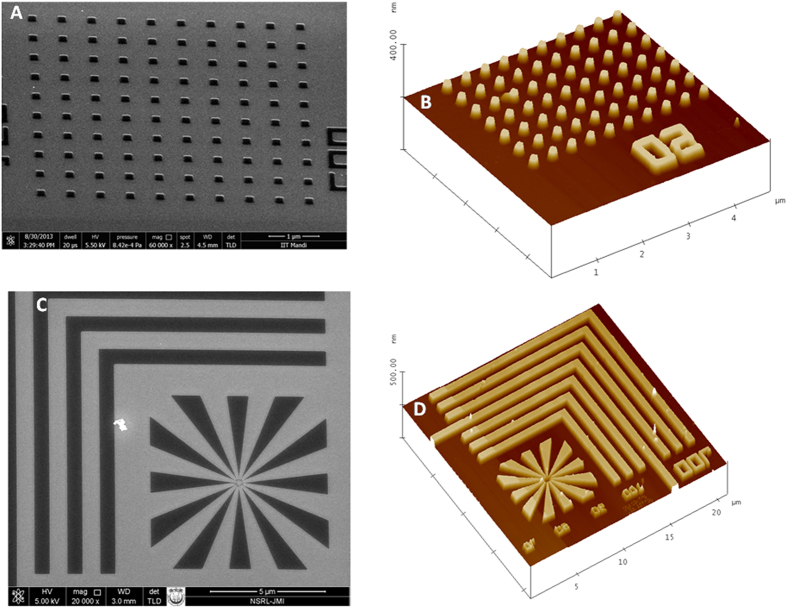
(**A**) FESEM images of EUV exposed nanopillars; (**B**) 3-D view of AFM images of nanopillars; (**C**) FESEM images of star-elbow features; (**D**) 3-D view of AFM images of star-elbow features. (EUV dose 88.19 mJ/cm^2^).

After FESEM characterization of these nanofeatures, AFM analyses were done to support our conclusion. The AFM integrated with a diamond needle (cantilever) tip was used for analyzing the features (DP15/Hi’Res-C/AIBS, MicroMasch, USA). The radius of curvature of the tip (apex-extratip) and spring (force) constant of the cantilever were 1 nm and 20 to 75 N/m (typical: 45 N/m), respectively. The resonant frequency of the used tips was between 265 and 400 kHz, typical: 325 kHz. Figure 6A,B represent the AFM images of nanodots from the surface 2.5 × 2.5 μm. The imaging was done with a slow scan speed in order to obtain good resolution and clarity. The AFM images revealed the presence of highly dense arrays of nanodots with feature size 34 nm. The three-dimensional images (Fig. 6B) demonstrate highly periodic arrays of nanodots with high aspect ratio. The variability in feature size of the patterns was established by AFM analysis (standard deviation was found to be approximately ±1). It is quite clear from the images that the patterns are fully developed up to the silicon surface. The AFM analysis also revealed that the patterned nanodots have good adhesion property with the silicon substrate. All these AFM imaging results clearly indicated that polyMAPDST can be considered as an excellent resist material for patterning dense periodic arrays of complex nanofeatures. Similarly, other features such as nanopillars and star-elbow have also been successfully patterned and fully characterized by FESEM/AFM analyses (Fig. 7).

#### Pattern Transfer

**Figure 8:**
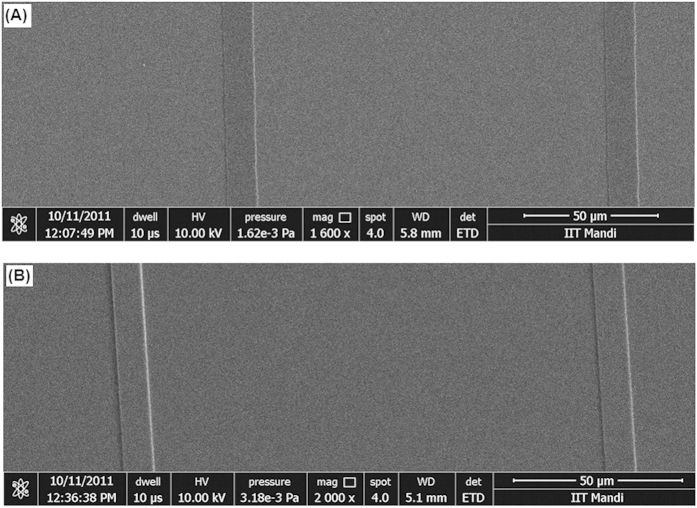
FESEM images of transferred patterns; (**A**) etch time 60 sec; (**B**) etch time 120 sec.

**Figure 9:**
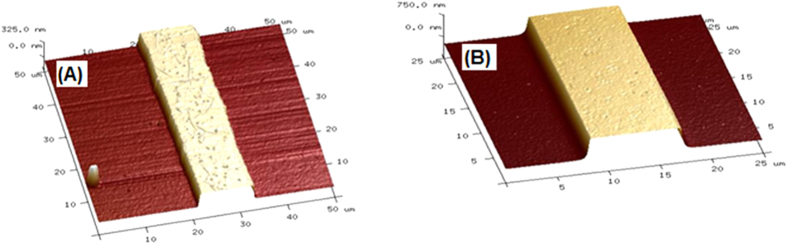
3-D AFM images of transferred patterns; (**A**) etch time 60 sec, 182 nm etch depth; (**B**) etch time 120 sec, 593 nm etch depth.

It has been well stated in the International Technology Roadmap for Semiconductors (ITRS)-2013 that in addition to high sensitivity and improved LER/LWR properties, one of the important criteria for new photoresists to be used for EUVL is to have high etch resistance in order to enable efficient pattern transfer using thinner films. PolyMAPDST was found to have very high etch resistance properties^23^, and the etch ratio with respect to the silicon substrate was found to be 7.2:1. This exceptionally high etch resistance, which is much higher than many commonly used organic photoresists^44^,^45^, encouraged us to investigate the pattern transfer into silicon substrate using ployMAPDST. To evaluate pattern transfer efficiency, polyMAPDST coated silicon substrates were subjected to e-beam lithography for generating line patterns onto silicon substrates. After developing, the patterned films were subjected to etching for different interval of time, 60 and 120 sec. Etching was done on a STSRIE tool using dry plasma etching technique, and the etch recipe involved SF_6_ with a flow rate of 5 sccm at a chamber pressure of 10 mTorr with an RF power of 20W. The patterned film thickness was measured before and after reactive ion etching follwed by removal of polymer film from the etched silicon surface (Table 1). These results indicated successful pattern trasfer into silicon substrate. The transferred patterns were fully characterized by FESEM and AFM measurements (Figs 8 and 9).

## Conclusions

To conclude, we investigated the potential of our recently developed negative tone non-chemically amplified photoresist polyMAPDST in patterning complex nanofeatures at 34 nm node and beyond. PolyMAPDST was synthesized as a polymeric material from the base monomer MAPDST which contains radiation sensitive triflate group. The integration of triflate group into polymer matrix made polyMAPDST radiation sensitive. The triflate unit underwent photodegradation upon exposure with high energy EUV photons, and thus, resulted in polarity switching in the exposed area of polymer matrix. Therefore, properly designed smart resist structure has been found to bring in desired polarity tuning without any external chemical amplification. The developed nanopatterns were observed to be highly ordered with negligible blurring of the pattern edge which is considered as an important factor in 3-D pattering. These wide-ranging complex features were characterized by FESEM and AFM techniques. The observations in FESEM were fully supported by AFM. Finally, and as a potential application, polyMAPDST has been successfully used for transferring patterns into silicon substrate using a compatible etch technique. Given the achievements in nanopatterning with sharp images, we strongly believe that the present methodology for featuring complex patterns will attract wide audience particularly in the area of micro/nanoelectronics.

## Methods

### General information

The monomer 4-(methacryloyloxy)phenyl)dimethylsulfonium triflate (MAPDST) for preparing poly(MAPDST) was synthesized following our recent reported procedure^21^. The polymerization of MAPDST was done using azobisisobutyronitrile (AIBN) initiated free radical polymerization reaction at 60 °C under dry nitrogen atmosphere for 2 days. The chemical structures of MAPDST and poly(MAPDST) were determined using FT-IR, ^1^H, ^13^C NMR and DSC-TGA analysis. The molecular weight (M_w_) was determined by Gel Permeation Chromatography (GPC), and found to be 20.7 × 10^3^. FT-IR spectra were recorded on a Perkin Elmer Spectrum 2 spectrophotometer, and ^1^H/^13^C NMR spectra on Jeol JNM ECX 500 MHz in deuterated dimethyl sulfoxide (DMSO-*d*_*6*_). TGA-DSC measurements were done on NETZSCH STA 449 F1 JUPITER Series instrument with a heating rate of 10 °C/min in N_2_ atmosphere over temperature range from 20 °C to 500 °C. Molecular weights and polydispersity (PDI) were determined by gel permeation chromatography (GPC) analysis using PL gel MIXED B & C 10 μm columns on a Agilent Technologies 1260 Infinity Series instrument.

### Resist formulation and EUV lithographic evaluation

Resist solution was prepared using 2% by weight of polymer in methanol. In order to remove larger particles, the solution was filtered through a 0.2 μm Teflon^®^ filter prior to application on the silicon substrate. The substrates were 2″ diameter p-type Si (100) wafers purchased from Wafer world, Inc. The silicon wafers were cleaned by RCA cleaning method to remove organic contaminants, prior to spinning. Then a dehydration bake was given to the cleaned silicon wafers at 200 °C for 10 min and cooled to room temperature. Thin films were prepared by spin coating the resist solution on a HMDS treated 200 mm silicon wafers for around 40 nm thickness and a soft-bake was given at 115 °C for 90 sec using a hotplate to remove the solvent completely from the resist film. The resulted thin films of photoresist were flood exposed with the respective E_0_ array using SEMATECH Berkeley Microfield Exposure Tool (MET). Post-exposure bake (PEB) was given at 100 °C for 90 sec to obtain good resist mask edges using hot plate and developed using standard concentration TMAH, 0.002 N aqueous solution by maintaining pH = 11.5 at room temperature for 15 sec. Finally, the developed patterns were rinsed with DI water for 8 sec and dried by passing pure nitrogen gas over the films.

### Imaging

The EUV exposed features were characterized by FESEM at energy of 5 keV. HRSEM images were taken using Nova Nano SEM 450 FEI instrument at JMI Central University and at IIT Mandi, India. In addition, the EUV features were characterized by AFM (Veeco-NanoScope IV, Multimode-AFM) providing 3D topographic images with height data. To obtain sharp and clearly defined sidewall patterns, high aspect ratio (HAR) tips were used (TESP-SS, Bruker AFM Probes, USA, tip height: 10–15 μm, tip radius: 2 nm, spring (force) constant of the cantilever 20 to 80 N∕m (typical: 42 N/m) and the resonant frequency between 230 and 410 kHz, nominal: 320 kHz). All images were acquired in a tapping mode, at UAM-I, Mexico and Indian Institute of Technology Mandi, India.

### Etch and pattern transfer

E-beam exposure was done on a Raith 150 lithography system at INUP, IIT Bombay. Resist solution was prepared using 4% by weight of polyMAPDST in methanol followed by filtration through a 0.2 μm Teflon^®^ filter prior to application. The films on silicon substrate were formed by spin coating with a spinning rate 3500 rpm for 60 sec. The film thickness was measured on a Bruker’s DektakXT™ (Santa Barbara, California) Stylus Profilometer. The films were exposed at a dose of 100 μC/cm^2^. The prebake and postbake were done for 90 sec at temperatures 115 °C and 100 °C respectively.

## Additional Information

**How to cite this article**: Ghosh, S. *et al*. Patterning highly ordered arrays of complex nanofeatures through EUV directed polarity switching of non chemically amplified photoresist. *Sci. Rep*. **6**, 22664; doi: 10.1038/srep22664 (2016).

## Figures and Tables

**Table 1:** Film thickness during etching followed by pattern transfer process (Bruker’s DektakXT™ Stylus Profilometer).

Before etchthickness (nm)	Etch time(second)	After etchthickness (nm)	Thickness after removal of polymerfilm from etched surface (nm)
103	60	350	307
118	120	698	645

## References

[b1] Martinez-Duart J. M., Martin-Palma R. J. & Agullo-Rueda F. Nanotechnology for Microelectronics and Optoelectronics (Elsevier, 2006).

[b2] Wolfgang F. Nanotechnology and Nanoelectronics: Materials, Devices, Measurement Techniques (Springer-Verlag Berlin Heidelberg, 2005).

[b3] Brian R. S. & Jeffrey T. G. Nanoelectronics: Nothing is like a vacuum. Nature Nanotechnology 7, 485–487 (2012). 10.1038/nnano.2012.130 22864166

[b4] Robert F. S. Optical Lithography Goes to Extremes–And Beyond. Science 293, 785–786 (2001). 11486068 10.1126/science.293.5531.785

[b5] Takashi I. & Shinji O. Pushing the limits of lithography. Nature 406, 1027–1031 (2000). 10984061 10.1038/35023233

[b6] Daniel P. S. Advances in Patterning Materials for 193 nm Immersion Lithography. Chem. Rev. 110, 321–360 (2010). 20070116 10.1021/cr900244n

[b7] Bernard F. Advanced optical lithography development, from UV to EUV. Microelectron. Eng. 61–62, 11–24 (2002).

[b8] Naulleau P. . The SEMATECH Berkeley MET pushing EUV development beyond 22-nm half pitch. Proc. SPIE 7636, 76361J (2010).

[b9] Anderson C. N. . The SEMATECH Berkeley MET: extending EUV learning down to 16nm half pitch. Proc. SPIE 7969, 79690R (2011).

[b10] Anderson C. N. . The SEMATECH Berkeley MET: demonstration of 15-nm half-pitch in chemically amplified EUV resist and sensitivity of EUV resists at 6.x-nm. Proc. SPIE 8322, 832212 (2012).

[b11] Seong-Yun M. & Jong-Man K. Chemistry of photolithographic imaging materials based on the chemical amplification concept. Journal of Photochemistry and Phobiology C: Photochemistry Reviews 8, 157–173 (2007).

[b12] Kimberly L. B., Kyle N. P., Qing Y. & Jeffrey S. M. Introduction to Photolithography: Preparation of Microscale Polymer Silhouettes. J. Chem. Educ. 82, 1365 (2005).

[b13] Pease R. F. & Chou S. Y. Lithography and other patterning techniques for future electronics. Proc. IEEE 96, 248–270 (2008).

[b14] Itani T. & Kozawa T. Resist Materials and Processes for Extreme Ultraviolet Lithography. Jap. J. Appl. Phys. 52, 010002 (2013).

[b15] Tsubaki H., Tarutani S., Inoue N., Takizawa H. & Goto T. EUV Resist Materials Design for 15nm Half Pitch and Below. J. Photopolym. Sci. Technol. 26, 649 (2013).

[b16] Kulshreshtha P. K. . Sub-20nm lithography negative tone chemically amplified resists using cross-linker additives. Proc. SPIE 8682, 86820N (2013).

[b17] Ma S., Con C., Yavuz M. & Cui B. Polystyrene negative resist for high-resolution electron beam lithography. Nanoscale Res. Lett. 6, 446 (2011). 21749679 10.1186/1556-276X-6-446 PMC3211865

[b18] Maruyama K. . Novel EUV resist materials and process for 20 nm half pitch and beyond. Proc. SPIE 8682, 86820B (2013).

[b19] Baek I. B. . Electron beam lithography patterning of sub-10nm line using hydrogen silsesquioxane for nanoscale device applications. J. Vac. Sci. Technol. B 23, 3120–3123 (2005).

[b20] Singh V., Satyanarayana V. S. V., Sharma S. K., Ghosh S. & Gonsalves K. E. Towards novel non-chemically amplified (n-CARS) negative resists for electron beam lithography applications. J. Mater. Chem. C 2, 2118–2122 (2014).

[b21] Satyanarayana V. S. V. . Radiation-sensitive novel polymeric resist materials: iterative synthesis and their EUV fragmentation studies. ACS Appl. Mater. Interfaces 6, 4223–4232 (2014). 24576018 10.1021/am405905p

[b22] Satyanarayana V. S. V. . A hybrid polymeric material bearing a ferrocene-based pendant organometallic functionality: synthesis and applications in nanopatterning using EUV lithography. RSC Advances 4, 59817–59820 (2014).

[b23] Singh V. . Performance evaluation of nonchemically amplified negative tone photoresists for e-beam and EUV lithography. Journal of Micro/Nanolithography, MEMS, and MOEMS 13, 043002–043002 (2014).

[b24] Kalyani V. . New Polyoxometalates Containing Hybrid Polymers and Their Potential for Nano-Patterning. Chem. Eur. J. 21, 2250–2258 (2015). 25431365 10.1002/chem.201405369

[b25] Bonam R., Verhagen P., Munder A. & Hartley J. Performance characterization of negative resists for sub-10-nm electron beam lithography. J. Vac. Sci. Technol. B 28, C6C34 (2010).

[b26] Peter I. T. . Synthesis and Properties of Diazopiperidiones for Use in Nonchemically Amplified Deep UV Photoresists. Chem. Mater. 16, 1770–1774 (2004).

[b27] Ramakrishnan G. & Jin-Baek K. Nonchemically amplified resists possessing cholate moiety for micropatterning of biomolecules. Microelectron. Eng. 88, 93–98 (2011).

[b28] Anguang Y. . Patterning of Tailored Polycarbonate Based Non-Chemically Amplified Resists Using Extreme Ultraviolet Lithography. Macromol. Rapid Commun. 31, 1449–1455 (2010). 21567550 10.1002/marc.201000117

[b29] Victor C. T. . Ultrasensitive non-chemically amplified low-contrast negative electron beam lithography resist with dual-tone behaviour. J. Mater. Chem. C 1, 1392–1398 (2013).

[b30] Gonsalves K. E. & Wu H. A Novel Single-Component Negative Resist for DUV and Electron Beam Lithography. Adv. Mater. 13, 195–197 (2001).

[b31] Wang M. . Incorporation of ionic photoacid generator (PAG) and base quencher into the resist polymer main chain for sub-50 nm resolution patterning. J. Mater. Chem. 18, 2704–2708 (2008).

[b32] Chen L. . Aqueous developable dual switching photoresists for nanolithography. J. Polym. Sci. A Polym. Chem. 50, 4255–4265 (2012).

[b33] Lawrie K. J. . Chain scission resists for extreme ultraviolet lithography based on high performance polysulfone-containing polymers. J. Mater. Chem. 21, 5629–5637 (2011).

[b34] Murillo R., Van Wolferen H. A., Abelmann L. & Lodder J. C. Fabrication of patterned magnetic nanodots by laser interference lithography. Microelectron. Eng. 78–79, 260–265 (2005).

[b35] Sumio H., Hirotaka S., Kazuo I. & Hayato S. Possibility to form an ultrahigh packed fine pit and dot arrays for future storage using EB writing. Microelectron. Eng. 83, 792–795 (2006).

[b36] Ankur V. & Ashutosh S. Sub-40 nm polymer dot arrays by self-organized dewetting of electron beam treated ultrathin polymer films. RSC Advances 2, 2247–2249 (2012).

[b37] Chen X., Palmer R. E. & Robinson A. P. G. A high resolution water soluble fullerene molecular resist for electron beam lithography. Nanotechnology 19, 275308 (2008). 21828704 10.1088/0957-4484/19/27/275308

[b38] Jochen G., Jurgen F. & Artur E. Time efficient fabrication of ultra large scale nano dot arrays using electron beam lithography. Microelectron. Eng. 97, 55–58 (2012).

[b39] Ebbesen T. W. . Extraordinary optical transmission through sub-wavelength hole arrays. Nature 391, 667–669 (1998).

[b40] Dana C. . Towards multiple readout application of plasmonic arrays. Beilstein J. Nanotechnol. 2, 501–508 (2011). 22003456 10.3762/bjnano.2.54 PMC3190620

[b41] Claudio D. R., Cheolmin P., Edwin L. T. & Bernard L. Microdomain patterns from directional eutectic solidification and epitaxy. Nature 405, 433–437 (2000). 10839533 10.1038/35013018

[b42] Samia B. A. . Electrocatalysis with monolayer modified highly organized macroporous electrodes. Electrochem. Commun. 5, 747–751 (2003).

[b43] Mathieu S. . Fabrication of highly ordered sub-20 nm silicon nanopillars by block copolymer lithography combined with resist design. J. Mater. Chem. C 1, 3544–3550 (2013).

[b44] Gibbons F. . Chemically amplified Fullerene Derivative molecular Electron Beam Resist. Small, 3, 2076–2080 (2007). 18008296 10.1002/smll.200700324

[b45] Jinxing Y. . Novel ester acetal polymers and their application for positive-tone chemically amplified i-line photoresists. J. Mater. Chem. C 1, 1160–11167 (2013).

